# Emergency paediatric critical care in England: describing trends using routine hospital data

**DOI:** 10.1136/archdischild-2019-317902

**Published:** 2020-05-22

**Authors:** Kate Marie Lewis, Sanjay M Parekh, Padmanabhan Ramnarayan, Ruth Gilbert, Pia Hardelid, Linda Wijlaars

**Affiliations:** 1 Population, Policy and Practice Research and Teaching Department, UCL Great Ormond Street Institute of Child Health, London, UK; 2 Children and Families Policy Research Unit, UCL Great Ormond Street Institute of Child Health, London, UK; 3 Children's Acute Transport Service, Great Ormond Street Hospital NHS Foundation Trust, London, UK; 4 Health Data Research UK, London, UK

**Keywords:** epidemiology, intensive care

## Abstract

**Objective:**

To determine trends in emergency admission rates requiring different levels of critical care in hospitals with and without a paediatric intensive care unit (PICU).

**Design:**

Birth cohort study created from Hospital Episode Statistics.

**Setting:**

National Health Service funded hospitals in England.

**Patients:**

8 577 680 singleton children born between 1 May 2003 and 31 April 2017.

**Outcome measures:**

Using procedure and diagnostic codes, we assigned indicators of high dependency care (eg, non-invasive ventilation) or intensive care (eg, invasive ventilation) to emergency admissions.

**Interventions:**

Children were followed up until their fifth birthday to estimate high dependency and intensive care admission rates in hospitals with and without a PICU. We tested the yearly trend of high dependency and intensive care admissions to hospitals without a PICU using logistic regression models.

**Results:**

Emergency admissions requiring high dependency care in hospitals without a PICU increased from 3.30 (95% CI 3.09 to 3.51) per 10 000 child-years in 2008/2009 to 7.58 (95% CI 7.28 to 7.89) in 2016/2017 and overtook hospitals with a PICU in 2015/2016. The odds of an admission requiring high dependency care to a hospital without a PICU compared with a hospital with a PICU increased by 9% per study year (OR 1.09, 95% CI 1.08 to 1.10). The same trend was not present for admissions requiring intensive care (OR 1.01, 95% CI 0.99 to 1.03).

**Conclusions:**

Between 2008/2009 and 2016/2017, an increasing proportion of admissions with indicators of high dependency care took place in hospitals without a PICU.

**What is already known on this topic?**

- A regional network of paediatric intensive care units (PICUs) was established in England in the 1990s to centralise care for critically ill children.
- Hospitals without a PICU continue to deliver high dependency care, but there is no national estimate of this activity.

**What this study adds?**

- From 2008 to 2016, the rate of paediatric admissions with indicators of high dependency care increased at a faster rate in hospitals without a dedicated PICU.
- This finding raises questions about the availability of resources to meet growing demands and highlights the need for standardised recording of critical care activities.

## Introduction

Paediatric critical care services deliver care to children who are acutely, seriously ill and need constant monitoring and medical intervention.^1^ Critical care can be categorised as *high dependency* (levels 1 and 2 of the UK’s three-tiered definition) for patients receiving organ support without invasive ventilation or *intensive care* (level 3) where patients require an even greater level of care—usually invasive ventilation or support for two or more organs and a higher nurse to patient ratio.^2 3^ In the UK National Health Service (NHS), paediatric intensive care was traditionally provided across a multitude of settings, including general wards and adult intensive care units.^4^ By the early 1990s, evidence of excess mortality among acutely ill children prompted concern about this fragmented model of paediatric care.^5–7^ This led to the centralisation of intensive care to dedicated centres with paediatric intensive care units (PICUs), and in 2002, the Paediatric Intensive Care Audit Network (PICANet) was established to monitor admissions to PICUs and their outcomes.^8^

Using data submitted by dedicated audit staff, PICANet monitors mortality and provides an annual overview of the variation in disease burden, mortality and staffing in the 32 PICUs across the UK and the Ireland.^8 9^ Information about high dependency care outside PICUs, however, relies on local audits and research studies.^2^ Regional audits have highlighted variation in the proportion of paediatric admissions to general hospitals that include high dependency care, ranging from 4.4% in the South West to 10.0% in Yorkshire. However, differences in the definition of high dependency care activity between audits limits both the comparability of audits across regions and their generalisability to the whole of England.^10–12^ Surveys using a national classification, the Paediatric Critical Care Minimum Dataset (PCCMDS), have also provided a snapshot of the increasing burden of high dependency care.^13 14^

Consistent collection and classification of high dependency care activity is needed to improve understanding of the burden of this type of care on hospitals and outcomes for the children who receive it. The aim of this study was to describe patterns of paediatric high dependency and intensive care among hospitals in England with and without a PICU using administrative hospital data.^15^ The objectives were: to explore the feasibility of using procedures and diagnostic codes recorded in routine hospital data as indicators of intensive and high dependency care and to determine whether trends over time in rates of emergency admissions requiring intensive and high dependency care among young children differ between hospitals with and without a PICU.

## Methods

### Data source and study population

Hospital Episode Statistics Admitted Patient Care (HES APC) records data on all admissions to NHS hospitals in England that require a hospital bed and includes both mothers’ delivery and babies’ birth admission records.^15^ Records have a pseudoanonymised ID (HESID) that can be used to link hospital records for the same individual over time.^15 16^ A cohort of all singleton births between 1 April 2003 and 31 March 2017 was created from HES APC data for this study. Babies from multiple births, stillborn babies and babies born to mothers not residing in England were excluded.^16^ The start of the study for each child was defined as 1 April 2008 or date of birth, whichever came last, and the end of the study as 31 March 2017, the child’s fifth birthday or date of death, whichever came first.

Using each child’s unique HESID cohort members were linked to any subsequent emergency hospital admissions between 1 April 2008 and 31 March 2017 that included at least one record indicating the provision of intensive or high dependency care, using the criteria set out below. Admissions were defined as consecutive episodes for the same individual within the same hospital and were excluded if: directly following a birth episode; occurring in the 7 days after birth; planned and not a transfer from a preceding emergency admission in another hospital; from a hospital with fewer than 52 emergency admissions for children aged 0–4 years per financial year; or from a maternity, community respite or specialist hospital. Hospitals with a PICU were defined using PICANet annual reports^17^ and manually identified from HES APC using the site of treatment or the provider code variable.^18 19^

### High dependency and intensive care indicators

Admissions were classified as high dependency or intensive care by matching PCCMDS procedures and diagnoses to relevant diagnosis, intervention and procedure codes in HES APC (see online supplementary table S1).^2 20^ Admissions with both levels of care were classified as intensive care. These indicators are not comprehensive for all intensive or high dependency care, but it was assumed they would be recorded consistently over time and could therefore be used to monitor trends. These indicators were validated against aggregate data on emergency admissions to PICUs provided by PICANet.

10.1136/archdischild-2019-317902.supp1 Supplementary data

### Risk factors

Date of admission was used to define year of event and age at admission (7 days–<6 months, 6 months–<1 year anad 1–4 years). The following risk factors were extracted from admission records at birth: sex (female or male); region of residence; child’s ethnicity; gestational age (preterm <37 weeks, term 37–41 weeks and post-term >41 weeks); birth weight (very low <1500 g, low 1500–2500 g, normal 2500–4200 g and above normal >4200 g); and the index of multiple deprivation (fifths). Presence of congenital anomalies (a dichotomous measure) was identified in hospital admissions or death certificates up to age 2 years using Hardelid *et al*’s chronic code list.^21^

### Statistical methods

Cohort characteristics were described by critical care admission status. Rates of emergency high dependency care and intensive care admissions in hospitals with and without a PICU were determined by dividing the number of admissions for each indicator by the person-time for all children at risk who were aged between 7 days and 4 completed years between 2008 and 2017. Multiple admissions for the same child were counted multiple times but with time spent in hospital not contributing to person-time. A conservative estimate of admissions to hospitals without a PICU was adopted by categorising any emergency admission that included a transfer from a hospital without a PICU to one with a PICU as occurring only in the hospital with a PICU. Admissions were measured per financial year, defined as 1 April–31 March of the next year.

The proportion of high dependency care and intensive care admissions that look took place outside a PICU was determined by dividing the number of admissions to hospitals without a PICU by the total number of admissions for each study year, stratified by level of care and age group. Among admissions with a high dependency or intensive care indicator, logistic regression was used to test the yearly trend of admission to hospitals without a PICU (as opposed to a hospital with a PICU). These results are presented overall and stratified by age group.

Sensitivity analysis was conducted to see if the results differed substantially when transfers were ignored and admissions categorised as belonging to the first hospital of emergency admission. To test the validity of these indicators, the number of emergency admissions with records of non-invasive and invasive ventilation to hospitals with a PICU in this dataset were compared with aggregate data provided by PICANet. Stata V.14.2^22^ was used for analyses and Microsoft Excel to produce graphs.

## Results

The study cohort consisted of 8 577 680, of which 48.7% were female, 64.1% were classified as having a white ethnicity and 33.4% were born to mothers residing in London or the South East (table 1; online supplementary figure S1). Average follow-up time per child was 4.16 years and 0.46% (n=39 024) of children had at least one emergency admission to hospital requiring high dependency or intensive care between 2008/2009 and 2016/2017. Emergency admissions for high dependency and intensive care contributed to 0.78% and 0.35% of all emergency admissions by children in the cohort, respectively. Compared with the whole cohort, children requiring critical care were more likely to have been born preterm (>18.1% vs 4.6%), weigh less than 1500 g at birth (>5.7% vs 0.6%) and have a congenital anomaly (>26.9% vs 3.0%). In total, there were 32 617 admissions involving high dependency care (with no intensive care) and 14 433 involving intensive care included in this study (table 2). The absolute number of admissions and length of stay for critical care admissions were higher for admissions to hospitals with a PICU compared with hospitals without a PICU.

**Table 1:** Birth characteristics of whole study cohort and of a subsample who had at least one emergency hospital admission* with indicators of high dependency care or intensive care between the ages of 7 days and 4 years

		Children n=8 577 680		Emergency admissions*			
				High dependency care		Intensive care	
		N	%	N	%	N	%
Emergency admission†	No	6 462 956	75.3	–		–	
	Yes	2 114 724	24.7	27 568	100.0	13 121	100.0
Sex	Female	4 174 749	48.7	11 894	43.1	5555	42.3
	Male	4 391 748	51.2	15 674	56.9	7566	57.7
	Missing	11 183	0.1	–	0.0	–	0.0
Ethnicity	White	5 496 696	64.1	19 100	69.3	8576	65.4
Asian or Asian British		770 991	9.0	3182	11.5	1768	13.5
Black or Black British		404 103	4.7	1290	4.7	822	6.3
	Mixed	322 563	3.8	977	3.5	544	4.2
	Other (any)	238 318	2.8	696	2.5	337	2.6
	Missing	1 345 009	15.7	2323	8.4	1074	8.2
Region	North East	385 335	4.5	1021	3.7	860	6.6
	North West	1 104 928	12.9	4412	16.0	1476	11.2
	Yorkshire	827 331	9.6	2474	9.0	1073	8.2
	East Midlands	667 681	7.8	3164	11.5	1448	11.0
	West Midlands	894 875	10.4	3817	13.8	1530	11.7
	East of England	881 488	10.3	2715	9.8	1493	11.4
	London	1 576 060	18.4	3643	13.2	2422	18.5
	South East	1 286 964	15.0	4040	14.7	1695	12.9
	South West	722 135	8.4	2182	7.9	1068	8.1
	Missing	230 883	2.7	100	0.4	56	0.4
IMD group	1 most deprived	2 334 812	27.2	9112	33.1	4395	33.5
	2	1 857 417	21.7	6188	22.4	3121	23.8
	3	1 539 676	17.9	4790	17.4	2176	16.6
	4	1 357 309	15.8	3800	13.8	1741	13.3
5 least deprived		1 301 427	15.2	3490	12.7	1586	12.1
	Missing	187 039	2.2	188	0.7	102	0.8
Gestational term (weeks)	Preterm (<37)	395 831	4.6	5003	18.1	2624	20.0
	Term (37–41)	6 221 403	72.5	17 679	64.1	8039	61.3
	Post-term (>41)	283 123	3.3	595	2.2	285	2.2
	Missing	1 677 323	19.6	4291	15.6	2173	16.6
Birth weight (kg)	Very low (<1.5)	52 274	0.6	1581	5.7	867	6.6
	Low (<2.5 g)	352 502	4.1	3200	11.6	1776	13.5
	Normal (2.5–4.2)	6 508 768	75.9	18 342	66.5	8300	63.3
Above normal (>4.2)		397 147	4.6	956	3.5	356	2.7
	Missing	1 266 989	14.8	3489	12.7	1822	13.9
Congenital anomalies	No	8 316 268	97.0	20 151	73.1	8099	61.7
	Yes	261 412	3.0	7417	26.9	5022	38.3

*Includes emergency admissions between 1 April 2008 and 31 March 2017. Only the first admission with an HDC or IC indicator per child is counted and where both indicators are present in the same admission, it is categorised as IC.

†Any emergency admission over the study period.

**Table 2:** Counts and rates (per 10 000 child-years) of admissions to hospital, by level of critical care, hospital type, year, age and sex of child: 2008/2009 to 2016/2017

High dependency care indicator*		Hospitals with a paediatric intensive care unit			Hospitals without a paediatric intensive care unit		
		N	Rate	95% CI	N	Rate	95% CI
Total		18 193	6.50	6.41 to 6.60	14 434	5.16	5.08 to 5.25
Year	2008/2009	1719	5.86	5.59 to 6.15	966	3.30	3.09 to 3.51
	2009/2010	1831	6.10	5.82 to 6.38	1328	4.42	4.19 to 4.67
	2010/2011	2067	6.73	6.45 to 7.03	1393	4.54	4.31 to 4.78
	2011/2012	2339	7.47	7.17 to 7.78	1450	4.63	4.40 to 4.88
	2012/2013	2289	7.19	6.90 to 7.49	1591	5.00	4.76 to 5.25
	2013/2014	2016	6.31	6.04 to 6.59	1713	5.36	5.12 to 5.62
	2014/2015	1974	6.20	5.93 to 6.48	1623	5.10	4.85 to 5.35
	2015/2016	1967	6.23	5.96 to 6.51	2002	6.34	6.07 to 6.63
	2016/2017	1991	6.38	6.10 to 6.66	2368	7.58	7.28 to 7.89
Age	7 days–<6 months	6402	22.66	22.11 to 23.22	8378	29.66	29.03 to 30.30
	6–<12 months	2606	9.22	8.87 to 9.58	1796	6.36	6.07 to 6.66
	1–4 years	9185	4.11	4.03 to 4.20	4260	1.91	1.85 to 1.97
Sex	Female	7899	5.80	5.68 to 5.93	6168	4.53	4.42 to 4.65
	Male	10 294	7.19	7.05 to 7.33	8266	5.77	5.65 to 5.90
**Percentile**		**25th**	**50th**	**75th**	**25th**	**50th**	**75th**
Length of stay (days)		4	8	17	2	4	7
**Intensive care indicator†**		**N**	**Rate**	**95% CI**	**N**	**Rate**	**95% CI**
Total		12 114	4.33	4.26 to 4.41	2309	0.83	0.79 to 0.86
Year	2008/2009	836	2.85	2.67 to 3.05	185	0.63	0.55 to 0.73
	2009/2010	1158	3.86	3.64 to 4.09	183	0.61	0.53 to 0.70
	2010/2011	1207	3.93	3.72 to 4.16	241	0.79	0.69 to 0.89
	2011/2012	1331	4.25	4.03 to 4.49	265	0.85	0.75 to 0.95
	2012/2013	1442	4.53	4.30 to 4.77	260	0.82	0.72 to 0.92
	2013/2014	1525	4.78	4.54 to 5.02	263	0.82	0.73 to 0.93
	2014/2015	1604	5.04	4.80 to 5.31	279	0.88	0.78 to 0.99
	2015/2016	1596	5.06	4.81 to 5.31	298	0.94	0.84 to 1.06
	2016/2017	1415	4.53	4.30 to 4.77	335	1.07	0.96 to 1.19
Age	7 days to <6 months	5454	19.31	18.81 to 19.83	877	3.11	2.91 to 3.32
	6 to <12 months	1785	6.32	6.03 to 6.62	296	1.05	0.94 to 1.17
	1 to 4 years	4875	2.18	2.12 to 2.25	1136	0.51	0.48 to 0.54
Sex	Female	5144	3.78	3.68 to 3.88	991	0.73	0.68 to 0.78
	Male	6970	4.87	4.75 to 4.98	1318	0.92	0.87 to 0.97
	**Percentile**	**25th**	**50th**	**75th**	**25th**	**50th**	**75th**
Length of stay (days)		4	9	18	1	2	4

*High dependency care includes non-invasive ventilation, central venous pressure monitoring, diabetic ketoacidosis, dialysis, intracranial pressure monitoring, cardiopulmonary resuscitation, arterial pressure monitoring, plasma filtration and red cell exchange.

†Intensive care includes invasive ventilation, endotracheal intubation, tracheostomy and extracorporeal membrane oxygenation.

**Figure 1:**
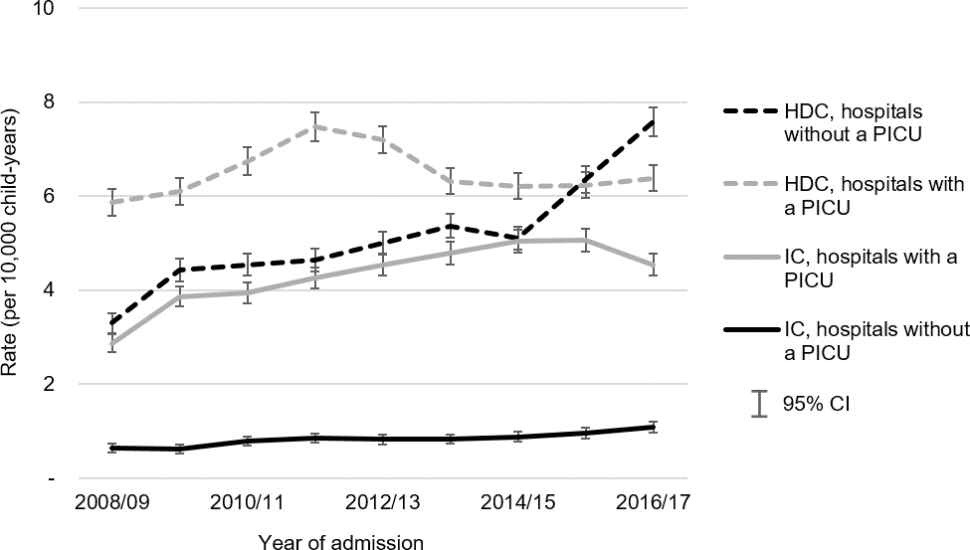
Rate (per 10 000 child-years) of emergency admissions with indicators of high dependency (HDC) or intensive care (IC) in children aged 7 days–4 years, by hospital type: 2008/2009 to 2016/2017. PICU, paediatric intensive care unit.

During the study period, the rate of emergency admissions requiring high dependency care in hospitals without a PICU more than doubled (figure 1), reaching 7.58 (95% CI 7.28 to 7.89) per 10 000 child-years in 2016/2017. In hospitals with a PICU, rates for the same type of care increased between 2008/2009 and 2011/2012 but then fell, culminating in a rate of 6.38 (95% CI 6.10 to 6.66) per 10 000 child-years in 2016/2017. Annual high dependency care admission rates rose by an average of 11.6% in hospitals without a PICU compared with 1.3% in hospitals with a PICU. The rate of high dependency care admissions in hospitals without a PICU overtook hospitals with a PICU in 2015/2016. Over the same period, the rate of emergency admissions requiring intensive care increased by an average of 7.3% in hospitals without a PICU, from 0.63 (95% CI 0.55 to 0.73) to 1.07 (95% CI 0.96 to 1.19) per 10 000 child-years, and 6.6% in hospitals with a PICU, from 2.85 (95% CI 2.67 to 3.05) to 4.53 (95% CI 4.30 to 4.77). Children aged less than 6 months and men had higher rates of high dependency and intensive care emergency admissions to hospitals with and without a PICU. Rates of admissions also varied by region, with the East Midlands having a particularly high rate of high dependency care admissions to hospitals with a PICU (see online supplementary table S2 and figure S2).

**Figure 2:**
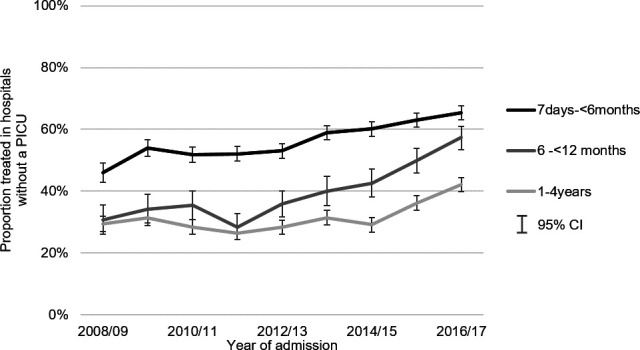
Proportion of all emergency admissions with an indicator of high dependency care treated at a hospital without a PICU, by age group and year of admission: 2008/2009 to 2016/2017. PICU, paediatric intensive care unit.

The proportion of emergency admissions with high dependency care that took place in hospitals without a PICU increased from 36.0% to 54.3% between 2008/2009 and 2016/2017 (see online supplementary table S3), a pattern replicated across all age groups (figure 2). The odds of admission to a hospital without compared with with a PICU for HDU care increased by 9% per study year (OR 1.09, 95% CI 1.08 to 1.10) (table 3). After stratifying by age group, this pattern was most pronounced in children aged between 6 and 12 months (OR 1.15, 95% CI 1.12 to 1.18). In contrast, the proportion of admissions requiring intensive care to a hospital without a PICU did not change substantially over the study period. The multivariable logistic regression models show very little change in the annual trend of admissions requiring intensive care in a hospital without compared with a hospital with a PICU (OR 1.01, 95% CI 0.99 to 1.03).

**Table 3:** ORs (95% CI) of receiving care in a hospital without a PICU* compared with a hospital with a PICU per 1 year increase, by level of critical care: 2008/2009 to 2016/2017

	High dependency care indicator		Intensive care indicator	
All ages (7 days to 4 years)	1.09	1.08 to 1.10	1.01	0.99 to 1.03
Age stratified				
7 days to <6 months	1.09	1.08 to 1.11	1.02	0.99 to 1.05
6 to <12 months	1.15	1.12 to 1.18	1.01	0.96 to 1.07
1 to 4 years	1.06	1.05 to 1.08	1.00	0.98 to 1.03

*Any emergency admission that included a transfer between types of hospitals was counted as an admission to a hospital with a PICU.

PICU, paediatric intensive care unit.

In sensitivity analyses that classified all emergency admissions as belonging to the first hospital of admission, the proportion of intensive care admissions treated at a hospital without a PICU rose from 16.0% to 31.2% (see online supplementary table S4). The annual trend in odds of admission for intensive care (OR 1.03, 95% CI 1.02 to 1.04) and high dependency care (OR 1.08, 95% CI 1.07 to 1.09) to hospitals without a PICU changed marginally. The number of admissions with invasive ventilation were 3–4 times higher in the PICANet dataset compared with those in HES APC across all study years (see online supplementary table S5, figure S3). The number of admissions with high dependency care was slightly underestimated in the HES APC dataset compared with PICANet (see online supplementary figure S4).

## Discussion

Between 2008/2009 and 2016/2017, the rate of high dependency care admissions rose by an average of 11.6% per year in hospitals without a PICU and overtook rates of comparable admissions in hospitals with a PICU (which rose by 1.3% per year) in 2015/2016. The annual increase in the proportion of high dependency care admissions treated in hospitals without a PICU was particularly pronounced in children aged 6 months to less than 1 year (OR 1.15, 95% CI 1.12 to 1.18). Rates of emergency admissions requiring intensive care rose at a slightly higher rate in hospitals without a PICU compared with hospitals with a PICU (7.3% compared with a 6.6% average annual increase) but remained fourfold higher in hospitals with a PICU across all study years.

Using a large-scale administrative hospital dataset, this study is the first to provide an estimate of nationally representative trends in incidence rates of emergency admissions requiring high dependency and intensive care across England. Paediatric critical care, unlike adult critical care, is not recorded in HES^15^; therefore, we developed indicators for high dependency care and intensive care using PCCMDS activities.^20^ However, using routinely collected data meant that our indicators were limited by the procedure and diagnosis codes recorded by hospitals, and, as shown in our comparison with PICANet data, it is likely that our study under-reports the prevalence of high dependency and intensive care.

The accuracy of HES APC data recording and the composition of procedures and diagnoses recorded has changed over time, especially with the advent of Payment by Results in 2004/2005.^15^ An indicator for invasive ventilation was only introduced in 2006, and its use may have grown more popular over time. In addition, invasive procedures such as heart surgery do not require invasive ventilation to also be recorded for a hospital to receive reimbursement, likely leading to lower rates of ventilation recording in these cases. We also note that PCCMDS includes several indicators that are not measured or difficult to capture in HES (eg, patient nursed in single occupancy cubicle). Linkage between the HES APC and PCCMDS datasets would help to address these shortcomings and improve future paediatric critical care research.

A study using PICANet data found that, between 2004 and 2013, admissions to PICUs requiring non-invasive ventilation almost doubled over the decade of study and rose faster than predicted based on population growth.^23^ Our findings suggest a slowing of this trend beyond 2013, while high dependency admissions to hospitals without a PICU continued to increase. Our study also points to an increasing trend of paediatric admissions requiring intensive care both in hospitals with and without a PICU; however, given the low absolute number of admissions of this type to hospitals without a PICU, we caution that further research is necessary to interpret this pattern. Increasing rates of admissions requiring critical care are likely driven, in part, by changes in the population, including increasing numbers of children with chronic and life-limiting illnesses and improved survival of infants born very preterm, combined with increasing levels of socioeconomic deprivation.^24–26^ Demographic characteristics may contribute to the noted geographical differences in admission rates, although variation in the infrastructure of local critical care provision also plays a role.^2 27^ For example, the South West has enhanced the capacity of regional hospitals to deliver paediatric non-invasive respiratory support, thus reducing the burden of high dependency care on hospitals with a PICU.^2^ Nottingham Children’s Hospital and Leicester Royal Infirmary, however, have PICUs colocated with paediatric high dependency units,^28 29^ likely contributing to higher rates of critical care in hospitals with a PICU in the East Midlands.

In 2017, the NHS England Paediatric Critical Care Review recommended that more high dependency care is delivered outside hospitals with a PICU to relieve these services of pressure and enable some children to be cared for closer to home.^30^ Our findings suggest that a shift in the delivery of high dependency care to hospitals without a PICU had already started by 2017. However, this trend raises questions about whether hospitals without a PICU are suitably equipped and have the staffing resources to meet this demand without compromising on the quality of care delivered. Since high dependency care is not recorded in HES APC, and hospitals without a PICU are not paid a top-up for providing critical care, our findings suggest that the Payment by Results system is currently under-reimbursing hospitals without a PICU that provide high dependency care. The Royal College of Paediatrics and Child Health has called for high dependency care to be given more focus, and previous studies have specifically emphasised training and organisational needs.^11^ Our study adds weight to these requests and highlights the need for improved and standardised recording of critical care activities so that this trend can be reliably monitored over time.^11^

## References

[1] NHS England Frequently asked questions, 2019 Available: https://www.england.nhs.uk/commissioning/spec-services/npc-crg/paediatric-critical-care-and-specialised-surgery-in-children-review/ [Accessed 13 Feb 2019].

[2] RCPCH High dependency care for children - time to move on. Royal College of Paediatrics and Child Health 2014.

[3] Shirley P Would you explain what the level 3 or level 2/3 ICU is? What is the difference between ICU and HDU? BMJ 2005;330:s184.3–s84. 10.1136/bmj.330.7499.s184-b

[4] Ramnarayan P, Polke E The state of paediatric intensive care retrieval in Britain. Arch Dis Child 2012;97:145–9. 10.1136/adc.2010.204503 21771765

[5] Pearson G, Shann F, Barry P, et al Should paediatric intensive care be centralised? Trent versus Victoria. Lancet 1997;349:1213–7. 10.1016/S0140-6736(96)12396-5 9130943

[6] Shann F Australian view of paediatric intensive care in Britain. Lancet 1993;342:68. 10.1016/0140-6736(93)91282-Q 8100909

[7] Ratcliffe J Provision of intensive care for children. A geographically integrated service may now be achieved. BMJ 1998;316:1547–8. 10.1136/bmj.316.7144.1547 9596587 PMC1113194

[8] PICANet About, 2018 Available: https://www.picanet.org.uk/ [Accessed 17 Aug 2019].

[9] Paediatric intensive care audit network annual report 2018 (published November 2018). Universities of Leeds and Leicester. Available: https://www.picanet.org.uk/annual-reporting-and-publications/ [Accessed 17 Feb 2019].

[10] Linnitt JDP, Walker J South West audit of critically ill children 10 year report: 2001-2010, 2010 Available: http://www.picanet.org.uk/research_projects.html [Accessed 01 Feb 2019].

[11] Linney MJ, Boyles C, Rutkowska H, et al Multi-centre study of paediatric high dependency activity. Arch Dis Child 2011;96:A89 10.1136/adc.2011.212563.207

[12] Rushforth K, Darowski M, McKinney PA Quantifying high dependency care: a prospective cohort study in Yorkshire (UK). Eur J Pediatr 2012;171:77–85. 10.1007/s00431-011-1475-9 21594577

[13] Campion-Smith J, Timperley S, Edees S G29(P) Quantifying paediatric high dependency care: does the paediatric critical care minimum dataset accurately capture workload? Arch Dis Child 2018;103:A12

[14] Morris KP, Oppong R, Holdback N, et al Defining criteria and resource use for high dependency care in children: an observational economic study. Arch Dis Child 2014;99:652–8. 10.1136/archdischild-2013-305133 24686043

[15] Herbert A, Wijlaars L, Zylbersztejn A, et al Data resource profile: Hospital episode statistics admitted patient care (HES APC). Int J Epidemiol 2017;46:1093–93i. 10.1093/ije/dyx015 28338941 PMC5837677

[16] Zylbersztejn A, Gilbert R, Hjern A, et al Child mortality in England compared with Sweden: a birth cohort study. Lancet 2018;391:2008–18. 10.1016/S0140-6736(18)30670-6 29731173 PMC5958228

[17] PICANet annual report Archive. Available: http://www.picanet.org.uk/Audit/Annual-Reporting/Annual-Report-Archive/ [Accessed 01 Feb 2019].

[18] NHS Digital Organisation data service. NHS digital. Available: https://digital.nhs.uk/organisation-data-service [Accessed 01 Feb 2019].

[19] CQC Care quality Commission. CQC, 2018 Available: http://www.cqc.org.uk/ [Accessed 01 Feb 2019].

[20] NHS Paediatric critical care minimum dataset. NHS data dictionary, 2018 Available: http://www.datadictionary.nhs.uk/data_dictionary/messages/supporting_data_sets/data_sets/paediatric_critical_care_minimum_data_set_fr.asp?shownav=1 [Accessed 01 Feb 2019].

[21] Royal College of Paediatrics and Child Health R Overview of child deaths in the four UK countries, 2013 Available: https://www.rcpch.ac.uk/resources/child-health-reviews-uk-retrospective-epidemiological-review-all-cause-mortality [Accessed 01 Feb 2019].

[22] Stata Statistical Software Release 14. [program]. College Station, TX: StataCorp LP, 2015.

[23] Davis P, Stutchfield C, Evans TA, et al Increasing admissions to paediatric intensive care units in England and Wales: more than just rising a birth rate. Arch Dis Child 2018;103:341–5. 10.1136/archdischild-2017-313915 29084723

[24] Wolfe I, Donkin A, Marmot M, et al UK child survival in a European context: recommendations for a national countdown collaboration. Arch Dis Child 2015;100:907–14. 10.1136/archdischild-2014-306752 25957319

[25] Knowles RL, Bull C, Wren C, et al Mortality with congenital heart defects in England and Wales, 1959–2009: exploring technological change through period and birth cohort analysis. Arch Dis Child 2012;97:861–5. 10.1136/archdischild-2012-301662 22753769

[26] Hardelid P, Dattani N, Gilbert R, et al Estimating the prevalence of chronic conditions in children who die in England, Scotland and Wales: a data linkage cohort study. BMJ Open 2014;4:e005331. 10.1136/bmjopen-2014-005331 PMC4127921 25085264

[27] Green CA, Yeates D, Goldacre A, et al Admission to hospital for bronchiolitis in England: trends over five decades, geographical variation and association with perinatal characteristics and subsequent asthma. Arch Dis Child 2016;101:140–6. 10.1136/archdischild-2015-308723 26342094 PMC4752648

[28] Nottingham University Hospitals NHS Trust Paediatric critical care unit [cited 2019 04 December], 2019 Available: https://www.nuh.nhs.uk/paediatric-critical-care-unit/

[29] University Hospitals of Leicester NHS Trust Paediatric intensive care unit, 2016 Available: http://www.eastmidlandscongenitalheart.nhs.uk/what-we-do/our-services/paediatric-intensive-care-unit/ [Accessed 04 Dec 2019].

[30] NHS England Paediatric critical care and specialised surgery in children review. NHS England; 2017; cited 2018 21 September. Available: https://www.england.nhs.uk/commissioning/spec-services/npc-crg/paediatric-critical-care-and-specialised-surgery-in-children-review/ [Accessed 13 Feb 2019].

