# Supplementary material for: Emergency paediatric critical care in England: describing trends using routine hospital data
Source: Arch Dis Child. 2020 May 22;105(11):1061–7. doi: 10.1136/archdischild-2019-317902 (PMC7588403; doi:10.1136/archdischild-2019-317902)
Supplement: Supplementary data [file archdischild-2019-317902supp001.pdf]

Supplementary material

Contents

Indicators of high dependency and intensive care (Table S1) 2

Flow chart (Figure S1) 4

Rates of admissions by region (Table S2, Figure S2) 5

Proportion of admissions treated at hospitals without a PICU (Tables S3) 7

Sensitivity analysis (Table S4) 9

PICANet comparison (Figure S3, Figure S4 and Table S5) 11

References 13

Lewis KM, *et al*. *Arch Dis Child* 2020; 105:1061–1067. doi: 10.1136/archdischild-2019-317902

Indicators of high dependency and intensive care (Table S1)

Section A and B of Table S1 identifies matching codes from the paediatric critical care minimum dataset (PCCMDS) and HES APC which were used to define admissions with high dependency or intensive care.[1] From the HES APC dataset, we identified procedure and diagnosis codes using the Office of Population Censuses and Survey Classification of Surgical Operations and Procedures (4th revision) (OPCS4) and International Statistical Classification of Diseases and Related Health Problems (10th revision, ICD-10) manuals, respectively.[23] Activities were classified as high dependency care or intensive care based on critical care Healthcare Resource Groups. Section C of Table S1 displays the PCCMDS activities that did not have a matching OPCS4 or ICD-10 code. These activities were excluded from the analysis.

Table S1. PCCMDS and OPCS4/ICD-10 codes* used to define (A) high dependency care and (B) intensive care; (C) displays unmatched codes

PCCMDS		OPCS4		
**Code/s**	**Description**	**Code/s***	**Description**	**N****
**A) High dependency care indicators**				
53	Non-invasive ventilation	E85.2	Non-invasive ventilation NEC (including continuous/intermittent positive airway pressure and negative pressure ventilation)	25,430
62	Central venous pressure monitoring	L91.1, L91.2, L91.3, L91.5	Insertion of / attention to central venous catheter NEC	12,285
70	Diabetic Ketoacidosis requiring continuous insulin infusion	E10.0, E10.1 (ICD-10 codes)	Insulin-dependent diabetes mellitus (including type I)/ ketosis-prone with coma with ketoacidosis; Insulin-dependent diabetes mellitus (including type I) / ketosis-prone with ketoacidosis (without coma)	1,887
05, 16, 66	Acute renal failure requiring dialysis; haemofiltration	X40.2, X40.3, X40.4	Peritoneal dialysis NEC, Haemodialysis NEC, Haemofiltration	1200
68, 69	Intracranial pressure monitoring; extra-ventricular drain	A20.3, A20.1	Monitoring of pressure in ventricle of brain; Drainage of ventricle of brain NEC	977
64	Cardiopulmonary Resuscitation (CPR) in the last 24hrs	X50.3	Advanced cardiac pulmonary resuscitation (CPR)	426
60	Invasive arterial monitoring	L72.2	Monitoring of arterial pressure	76
67	Plasmafiltration	X32.2, X32.3, X32.4, X32.5	Exchange of plasma	90
4	Exchange transfusion	X32.6	Red cell exchange	24

**B) Intensive care indicators**				
51, 52	Invasive ventilation via endotracheal tube; Invasive ventilation via tracheostomy tube	E85.1, X56.2, E42.3	Invasive Ventilation; Endotracheal intubation; Temporary tracheostomy	14,392
65	Extracorporeal membrane oxygenation (ECMO); Ventricular assist device (VAD); aortic balloon pump (ABP)	X58.1	ECMO	266

C) PCCMDS codes not matched to HES data

Code/s	Description
06	Continuous infusion of inotrope, pulmonary vasodilator or prostaglandin (PATIENT received a continuous infusion of an inotrope, vasodilator (includes pulmonary vasodilators) or prostaglandin)
13	Tracheostomy cared for by nursing staff (PATIENT receiving care of tracheostomy cared for by nursing staff not by an external Carer (e.g. parent))
55	Nasopharyngeal airway
56	Advanced ventilatory support (Jet or Oscillatory ventilation)
57	Upper airway obstruction requiring nebulised Epinephrine/ Adrenaline
58	Apnoea requiring intervention
59	Acute severe asthma requiring intravenous bronchodilator therapy or continuous nebuliser
61	Cardiac pacing via an external box (pacing wires or external pads or oesophageal pacing)
63	Bolus intravenous fluids (> 80 ml/kg/day) in addition to maintenance intravenous fluids
71	Intravenous infusion of thrombolytic agent (limited to tissue plasminogen activator [tPA] and streptokinase)
74	PATIENT nursed in single occupancy cubicle
80	Heated Humidified High Flow Therapy (HHHFT) (PATIENT receiving HHHFT)
85	PATIENT has an epidural catheter in situ
94	PATIENT has arrhythmia requiring intravenous anti-arrhythmic therapy
95	PATIENT has reduced conscious level (Glasgow Coma Score 12 or below) and hourly (or more frequent) Glasgow Coma Score monitoring
96	Intravenous infusion of sedative agent (PATIENT receiving continuous intravenous infusion of sedative agent)
97	PATIENT has status epilepticus requiring treatment with continuous intravenous infusion

*OPCS4 codes presented unless otherwise stated

**Frequency of indicators in final dataset – groups are not mutually exclusive

Flow chart (Figure S1)

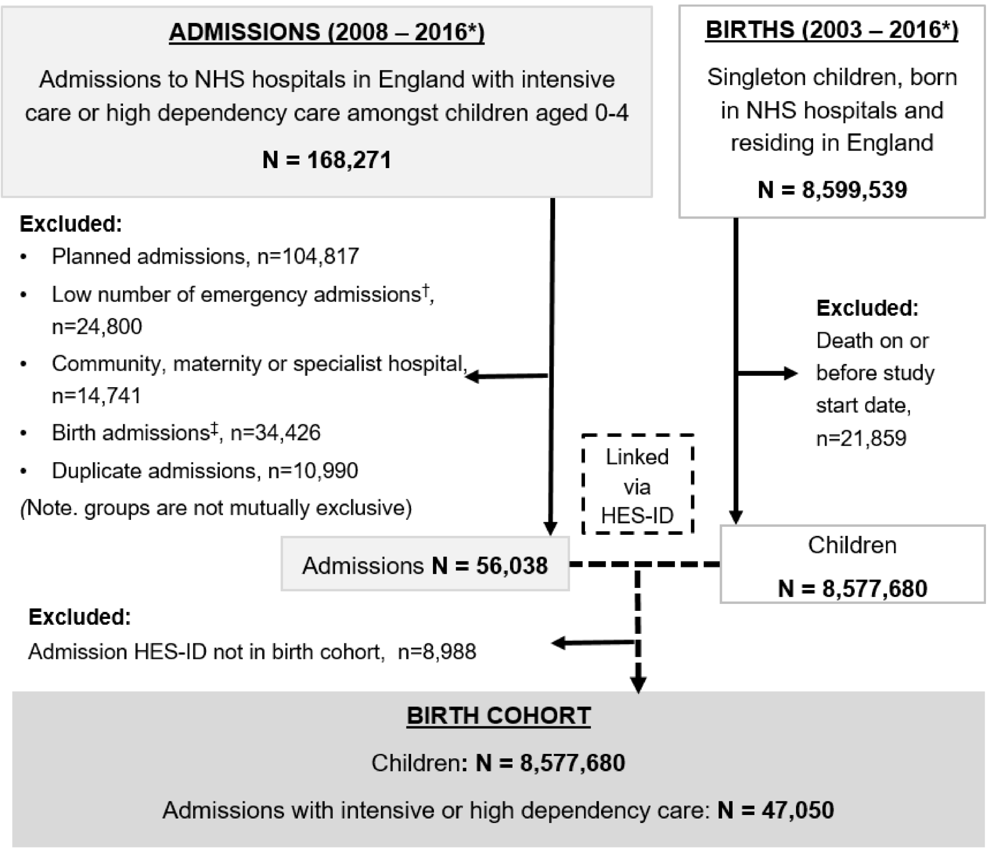

* *Refers to financial years (April to March)*

† *Hospital sites-years with less than 52 emergency episodes per year was deleted (if a hospital site had years with more than 52 emergency episodes per year, these were included)*

‡ *episodes that were less than 7 days after date of birth (if length of post-natal stay was <u><</u>5 days) or less than two days after date of discharge from birth admission (if length of post-natal stay was <u>></u>6 days)*

Figure S1. Flow diagram illustrating study cohort and admission selection

Lewis KM, *et al. Arch Dis Child* 2020; 105:1061–1067. doi: 10.1136/archdischild-2019-317902

Rates of admissions by region (Table S2, Figure S2)

Table S2. Counts and rates (per 10,000 child-years) of admissions to hospital, by level of care, hospital type and region of residence at birth: 2008/09 to 2016/17

		Hospitals with a PICU			Hospitals without a PICU		
**High dependency care***		**N**	**Rate**	**(95% CI)**	**N**	**Rate**	**(95% CI)**
Region	North East	848	6.71	(6.27, 7.17)	393	3.11	(2.82, 3.43)
	North West	3,148	8.71	(8.41, 9.02)	2,001	5.54	(5.30, 5.79)
	Yorkshire and Humber	2,146	7.97	(7.64, 8.31)	769	2.86	(2.66, 3.06)
	East Midlands	2,910	13.26	(12.79, 13.75)	827	3.77	(3.52, 4.04)
	West Midlands	2,386	8.23	(7.90, 8.56)	2,261	7.80	(7.48, 8.13)
	East of England	963	3.36	(3.15, 3.58)	2,142	7.46	(7.15, 7.79)
	London	2,381	4.59	(4.41, 4.78)	1,935	3.73	(3.57, 3.90)
	South East	2,237	5.32	(5.10, 5.55)	2,524	6.00	(5.77, 6.24)
	South West	1,101	4.68	(4.41, 4.96)	1,536	6.53	(6.21, 6.86)
	Unknown	73	1.04	(0.83, 1.31)	46	0.66	(0.49, 0.87)
**Intensive care****		**N**	**Rate**	**(95% CI)**	**N**	**Rate**	**(95% CI)**
Region	North East	895	7.08	(6.63, 7.56)	54	0.43	(0.33, 0.56)
	North West	1,267	3.51	(3.32, 3.71)	329	0.91	(0.82, 1.01)
	Yorkshire and Humber	980	3.64	(3.42, 3.87)	190	0.71	(0.61, 0.81)
	East Midlands	1,460	6.66	(6.32, 7.01)	127	0.58	(0.49, 0.69)
	West Midlands	1,563	5.39	(5.13, 5.67)	143	0.49	(0.42, 0.58)
	East of England	1,415	4.93	(4.68, 5.20)	226	0.79	(0.69, 0.90)
	London	2,448	4.72	(4.54, 4.91)	251	0.48	(0.43, 0.55)
	South East	1,263	3.00	(2.84, 3.17)	611	1.45	(1.34, 1.57)
	South West	769	3.27	(3.04, 3.51)	370	1.57	(1.42, 1.74)
	Unknown	54	0.77	(0.58, 1.00)	<10	0.11	(0.06, 0.23)

*High dependency care includes non-invasive ventilation; central venous pressure monitoring; diabetic ketoacidosis; dialysis; intracranial pressure monitoring; cardiopulmonary resuscitation; arterial pressure monitoring; plasma filtration; red cell exchange; **Intensive care includes invasive ventilation; endotracheal intubation; tracheostomy; extracorporeal membrane oxygenation.

Lewis KM, *et al. Arch Dis Child* 2020; 105:1061–1067. doi: 10.1136/archdischild-2019-317902

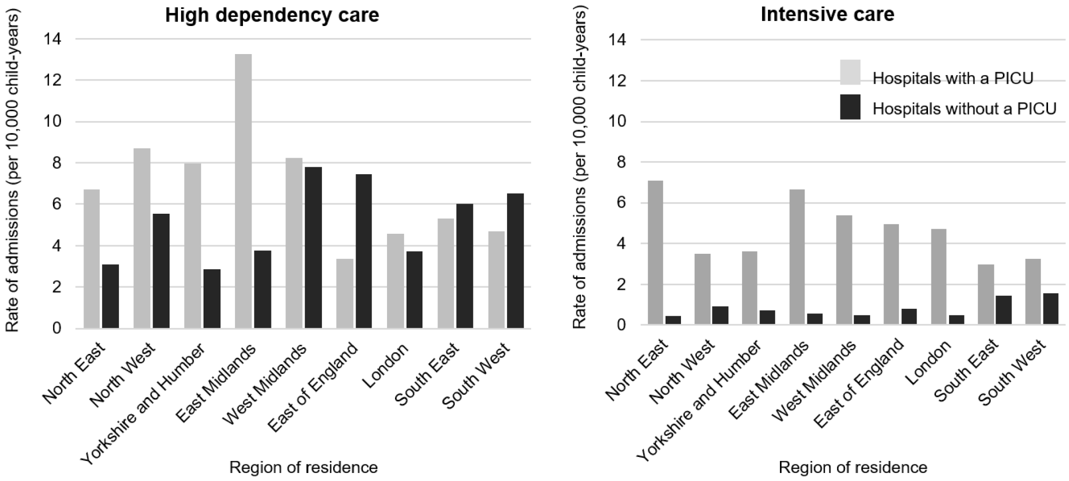

Figure S2. Rates (per 10,000 child-years) of admissions to hospital, by level of care, hospital type and region of residence at birth: 2008/09 to 2016/17

Lewis KM, *et al. Arch Dis Child* 2020; 105:1061–1067. doi: 10.1136/archdischild-2019-317902

Proportion of admissions treated at hospitals without a PICU (Tables S3)

Table S3. Total number of emergency critical care admissions and proportion (95% CI) treated at a hospital without a PICU, by age of patient and critical care indicator: England, 2008/09 to 2016/17

Year	High dependency care indicator			Intensive care indicator		
	Total N	% at hospital without a PICU (95% CI)		Total N	% at hospital without a PICU (95% CI)	
All ages (7 days to 4 years)						
2008/09	2,685	36.0	(34.2, 37.8)	1,021	18.1	(15.8, 20.6)
2009/10	3,159	42.0	(40.3, 43.8)	1,341	13.6	(11.9, 15.6)
2010/11	3,460	40.3	(38.6, 41.9)	1,448	16.6	(14.8, 18.7)
2011/12	3,789	38.3	(36.7, 39.8)	1,596	16.6	(14.8, 18.5)
2012/13	3,880	41.0	(39.5, 42.6)	1,702	15.3	(13.6, 17.1)
2013/14	3,729	45.9	(44.4, 47.6)	1,788	14.7	(13.1, 16.4)
2014/15	3,597	45.1	(43.5, 46.8)	1,883	14.8	(13.2, 16.5)
2015/16	3,969	50.4	(48.9, 52.0)	1,894	15.7	(14.1, 17.5)
2016/17	4,359	54.3	(52.8, 55.8)	1,750	19.1	(17.3, 21.1)
**Overall**	32,627	44.2	(43.7, 44.8)	14,423	16.0	(15.4, 16.6)
7 days to <6 months						
2008/09	1,043	46.0	(43.0, 49.1)	467	18.2	(14.8, 22.0)
2009/10	1,438	54.0	(51.5, 56.8)	587	10.7	(8.3, 13.5)
2010/11	1,622	51.8	(49.4, 54.3)	688	14.0	(11.5, 16.8)
2011/12	1,706	52.1	(49.7, 54.5)	712	12.5	(10.2, 15.2)
2012/13	1,837	53.0	(50.7, 55.5)	701	13.7	(11.2, 16.5)
2013/14	1,834	59.0	(56.8, 61.4)	772	12.0	(9.8, 14.6)
2014/15	1,655	60.1	(56.7, 61.3)	837	11.9	(9.8, 14.3)
2015/16	1,795	63.1	(60.8, 65.3)	804	14.2	(11.8, 16.8)
2016/17	1,850	65.4	(63.1, 67.5)	763	18.5	(15.8, 21.4)
**Overall**	14,780	56.7	(55.9, 57.5)	6331	13.9	(13.0, 14.7)
6 months to <1 year)						
2008/09	376	30.6	(26.0, 35.5)	127	18.9	(12.5, 26.8)
2009/10	406	34.2	(29.6, 39.1)	197	11.2	(7.1, 16.4)
2010/11	436	35.3	(30.8, 40.0)	205	14.6	(10.1, 20.2)
2011/12	482	28.4	(24.4, 32.7)	231	16.0	(11.5, 21.4)
2012/13	511	35.8	(31.6, 40.1)	264	9.5	(6.2, 13.7)
2013/14	422	40.0	(35.3, 44.9)	268	14.9	(10.9, 19.8)
2014/15	470	42.6	(38.1, 47.2)	248	12.5	(8.7, 17.3)
2015/16	613	49.9	(45.9, 54.0)	300	16.3	(12.3, 21.0)

Lewis KM, *et al. Arch Dis Child* 2020; 105:1061–1067. doi: 10.1136/archdischild-2019-317902

2016/17	686	57.3	(53.5, 61.0)	241	15.8	(11.4, 21.0)
**Overall**	4402	40.8	(39.3, 42.3)	2081	14.2	(12.8, 15.8)
1 to 4 years						
2008/09	1,266	29.3	(26.8, 31.9)	427	17.8	(14.3, 21.7)
2009/10	1,315	31.4	(28.9, 34.0)	557	17.6	(14.5, 21.1)
2010/11	1,402	28.4	(26.0, 30.8)	555	20.7	(17.4, 24.3)
2011/12	1,601	26.5	(24.3, 28.7)	653	21.3	(18.2, 24.6)
2012/13	1,532	28.3	(26.1, 30.7)	737	18.9	(16.1, 21.9)
2013/14	1,473	31.4	(29.0, 33.8)	748	17.4	(14.7, 20.3)
2014/15	1,472	29.1	(26.8, 31.5)	798	18.5	(15.9, 21.4)
2015/16	1,561	36.1	(33.7, 38.6)	790	17.1	(14.5, 19.9)
2016/17	1,823	42.0	(39.7, 44.3)	746	20.9	(18.0, 24.0)
**Overall**	13,445	31.7	(30.9, 32.5)	6011	18.9	(17.9, 19.9)

Lewis KM, *et al. Arch Dis Child* 2020; 105:1061–1067. doi: 10.1136/archdischild-2019-317902

Sensitivity analysis (Table S4)

Table S4. Proportion (95% CI) of all emergency critical care admissions treated at a hospital without a PICU*, by critical care indicator and age of patient: England, 2008/09 to 2016/17

	**All ages (7 days-<4 years)**		**7 days-<6 months**		**6-<12 months**		**1-4 years**	
**Year**	**%**	**(95% CI)**	**%**	**(95% CI)**	**%**	**(95% CI)**	**%**	**(95% CI)**
**High dependency care indicator**								
2008/09	39.2	(37.3, 41.1)	40.1	(38.1, 42.1)	35.1	(28.6, 42.2)	32.4	(25.5, 39.8)
2009/10	46.1	(44.3, 47.8)	47.4	(45.5, 49.3)	33.8	(27.4, 40.7)	40.4	(33.5, 47.6)
2010/11	44.3	(42.6, 46.0)	44.8	(43.0, 46.6)	46.3	(40.0, 52.8)	33.9	(27.2, 41.0)
2011/12	42.3	(40.7, 43.9)	43.6	(41.9, 45.3)	37.8	(32.0, 43.9)	27.6	(21.7, 34.1)
2012/13	45.0	(43.4, 46.6)	45.9	(44.2, 47.6)	39.9	(34.2, 45.7)	38.8	(32.5, 45.4)
2013/14	49.8	(48.2, 51.4)	50.5	(48.8, 52.2)	44.6	(38.3, 51.0)	44.2	(36.6, 51.9)
2014/15	48.3	(46.6, 49.9)	48.7	(46.9, 50.4)	50.8	(44.5, 57.1)	40.1	(33.7, 46.8)
2015/16	53.7	(52.1, 55.2)	53.9	(52.2, 55.6)	52.4	(47.1, 57.8)	52.9	(46.8, 58.8)
2016/17	57.2	(55.7, 58.7)	56.7	(55.1, 58.3)	65.6	(60.6, 70.3)	52.3	(46.5, 58.1)
**Overall**	**47.8**	**(47.3, 48.4)**	**48.4**	**(47.8, 49.0)**	**46.9**	**(44.9, 48.9)**	**41.3**	**(39.2, 43.5)**
**Intensive care indicator**								
2008/09	28.1	(25.4, 31.0)	28.3	(25.3, 31.4)	27.7	(17.3, 40.2)	26.6	(16.3, 39.1)
2009/10	25.8	(23.5, 28.3)	26.5	(24.0, 29.2)	24.2	(16.2, 33.9)	19.4	(12.3, 28.4)
2010/11	29.6	(27.3, 32.1)	29.9	(27.3, 32.5)	29.9	(21.0, 40.0)	27.0	(19.1, 36.0)
2011/12	31.5	(29.2, 33.8)	31.4	(29.0, 34.0)	31.6	(23.3, 40.9)	31.6	(23.2, 40.9)
2012/13	31.8	(29.6, 34.1)	32.8	(30.4, 35.4)	28.7	(21.4, 36.8)	24.0	(16.8, 32.5)
2013/14	32.3	(30.1, 34.5)	32.0	(29.6, 34.4)	35.8	(28.1, 44.1)	32.2	(24.0, 41.3)
2014/15	30.7	(28.6, 32.8)	31.6	(29.3, 33.9)	22.0	(15.2, 30.3)	27.7	(19.9, 36.7)
2015/16	32.4	(30.3, 34.6)	32.1	(29.8, 34.5)	35.8	(28.1, 44.1)	31.7	(24.3, 40.0)
2016/17	35.8	(33.6, 38.2)	36.4	(33.9, 38.9)	33.1	(25.4, 41.6)	31.4	(22.5, 41.3)
**Overall**	**31.2**	**(30.5, 32.0)**	**31.5**	**(30.7,**	**30.4**	**(27.6, 33.2)**	**28.2**	**(25.4, 31.1)**

		**32.4)**

* Transfers ignored and admissions categorised as belonging to the first hospital of emergency admission.

Lewis KM, *et al. Arch Dis Child* 2020; 105:1061–1067. doi: 10.1136/archdischild-2019-317902

PICANet comparison (Figure S3, Figure S4 and Table S5)

Table S5, below, presents the number of emergency admissions to PICUs (or hospitals with a PICU) requiring non-invasive and invasive ventilation as recorded by PICANet and HES. The data is presented by financial year from 2008/09 to 2016/17 for children 7 days to 4 years old. All PICUs/hospitals with a PICU in England are included except those in the East of England region.

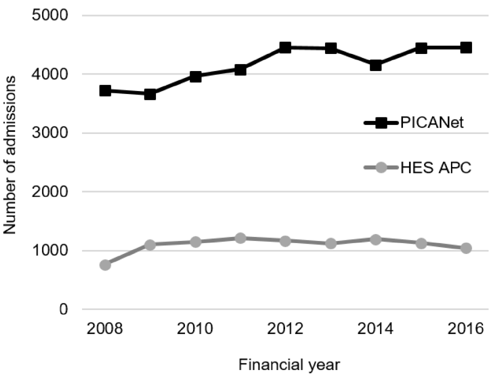

Figure S3. Emergency admissions requiring invasive ventilation, by financial year and dataset: Children 7 days-4 years old

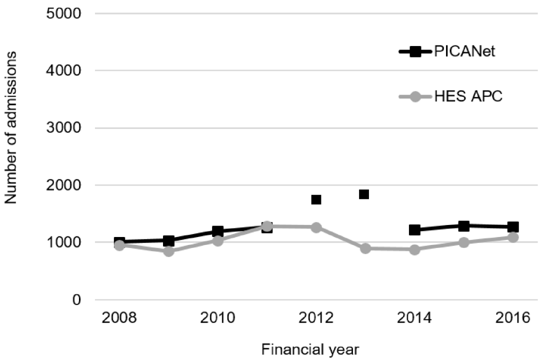

Figure S4. Emergency admissions requiring non-invasive ventilation, by financial year and dataset: Children 7 days-4 years old; black squares outside of PICANet time series represent recording error (as explained in text)

Invasive ventilation

The number of admissions with invasive ventilation were 3 to 4 times higher in the PICANet dataset compared to those in HES, as shown in Figure S3.

Non-invasive ventilation

The number of admissions with non-invasive ventilation in the PICANet dataset increased substantially in 2012 and 2013, whilst the same indicator in the HES dataset did not. As confirmed by PICANet, these anomalies were to PICUs mistakenly recording a new intervention (high-flow nasal cannula therapy) as non-invasive. As stated in PICANet's 2014 and 2015 annual reports,[4] the decision to record this treatment as supplemental oxygen therapy (irrespective of ventilatory state) *not* non-invasive ventilation was clarified in December 2013. There is no specific code for high flow nasal

oxygen in HES so we were unable to account for this discrepancy in our dataset, and therefore excluded 2012/13 and 2013/14 from the non-invasive ventilation comparison.

Ignoring this inconsistency, the number of non-invasive ventilation admissions appear broadly similar across the two datasets between 2008/09 and 2011/12 and between 2014/15 and 2016/17 (Figure S4). PICANet data only provides high dependency care information for patients whilst they are in the PICU, whereas our HES derived indicators cannot differentiate between care performed within the PICU or anywhere else in a tertiary hospital. Thus our dataset is underreporting the number of high dependency care admissions.

Table S5. Number of emergency admissions amongst children 7 days-4 years old requiring non-invasive and invasive ventilation, by financial year and dataset: England (excluding East of England region), 2008/09 to 2019/17

	Invasive ventilation		Non-invasive ventilation	
**Financial year**	PICANet	HES APC*	PICANet	HES APC**
**2008**	3726	763	1010	955
**2009**	3665	1102	1037	850
**2010**	3968	1151	1198	1033
**2011**	4085	1214	1266	1287
**2012**	4459	1167	1711	1270
**2013**	4442	1123	1842	898
**2014**	4161	1197	1224	877
**2015**	4451	1130	1291	1002
**2016**	4460	1047	1272	1091

*OPSC code E85.2; **OPSC code E85.1, X56.2 or E42.3

References

1. NHS. Paediatric Critical Care Minimum Dataset. NHS Data Dictionary; 2018. Available from: http://www.datadictionary.nhs.uk/data_dictionary/messages/supporting_data_sets/data_sets/paediatric_critical_care_minimum_data_set_fr.asp?shownav=1 accessed 1 February 2019.

2.NHS Digital. NHS Classifications OPCS-4. NHS Digital. Available from: https://isd.digital.nhs.uk/trud3/user/guest/group/0/pack/10 accessed 1 February 2019.

3. WHO. International Statistical Classification of Disease and Related Health Problems 10th Revision (ICD-10). World Health Organisation (WHO); 2016. Available from: http://apps.who.int/classifications/icd10/browse/2010/en accessed 1 February 2019.

4. PICANet. About. 2018. Available from: https://www.picanet.org.uk/ accessed 17 August 2018.
